# Monitoring Flexions and Torsions of the Trunk via Gyroscope-Calibrated Capacitive Elastomeric Wearable Sensors

**DOI:** 10.3390/s21206706

**Published:** 2021-10-09

**Authors:** Gabriele Frediani, Federica Vannetti, Leonardo Bocchi, Giovanni Zonfrillo, Federico Carpi

**Affiliations:** 1 Department of Industrial Engineering, University of Florence, 50121 Florence, Italy; gabriele.frediani@unifi.it (G.F.); giovanni.zonfrillo@unifi.it (G.Z.); 2 IRCCS Fondazione don Carlo Gnocchi ONLUS, 50143 Florence, Italy; fvannetti@dongnocchi.it; 3 Department of Information Engineering, University of Florence, 50121 Florence, Italy; leonardo.bocchi@unifi.it

**Keywords:** capacitive, elastomer, flexion, torsion, sensor, wearable, wireless

## Abstract

Reliable, easy-to-use, and cost-effective wearable sensors are desirable for continuous measurements of flexions and torsions of the trunk, in order to assess risks and prevent injuries related to body movements in various contexts. Piezo-capacitive stretch sensors, made of dielectric elastomer membranes coated with compliant electrodes, have recently been described as a wearable, lightweight and low-cost technology to monitor body kinematics. An increase of their capacitance upon stretching can be used to sense angular movements. Here, we report on a wearable wireless system that, using two sensing stripes arranged on shoulder straps, can detect flexions and torsions of the trunk, following a simple and fast calibration with a conventional tri-axial gyroscope on board. The piezo-capacitive sensors avoid the errors that would be introduced by continuous sensing with a gyroscope, due to its typical drift. Relative to stereophotogrammetry (non-wearable standard system for motion capture), pure flexions and pure torsions could be detected by the piezo-capacitive sensors with a root mean square error of ~8° and ~12°, respectively, whilst for flexion and torsion components in compound movements, the error was ~13° and ~15°, respectively.

## 1. Introduction

In order to prevent and assess the risk of musculoskeletal injuries related to body movements in various contexts, continuous measurements of flexions and torsions of the human trunk are desirable. Indeed, a variety of jobs expose workers to incorrect and even risky body postures and movements, characterized by excessive and/or repeated flexions and/or torsions of the trunk. Accordingly, the movements of the trunk are usually adopted as key indicators to assess risks in occupational health management [1,2,3,4]. Therefore, the possibility of continuously monitoring, in a simple way, flexions and torsions of the trunk could improve health management programs in workplaces. Especially, such measurements should preferably be obtained via wearable sensors, ideally able to combine comfort, ease of use, reliability and low cost.

Today, the gold standard to monitor human kinematics is represented by stereophotogrammetry, where markers arranged on the subject are tracked by external cameras [5]. The high accuracy of this methodology is counterbalanced by the need for bulky, complex-to-use and expensive equipment, which also requires that the subject is confined within an empty space, so as to enable continuous tracking by the cameras. As a result, in several contexts, stereophotogrammetry is not usable, and wearable solutions [6] are preferable/necessary. Among them, the most sensitive and most used are inertial measurement units (IMUs), electrogoniometers and electromagnetic sensors, as briefly recalled below.

IMU devices host tri-axial accelerometers, gyroscopes and magnetometers within a small integrated unit. The accuracy of accelerometers and gyroscopes is respectively limited by sudden accelerations/decelerations and drifts (resulting from the integration of an angular velocity), leading to errors that frequently require mutual compensations, also in combination with the co-located magnetometer [7,8,9]. The resulting sensory fusion complicates the sensing algorithms and, in the case of the use of the magnetometer, also exposes the measurements to electromagnetic interference. Additional complexity and computational costs often come from the combination with biomechanical models, which are adopted to resolve uncertainties and therefore improve the accuracy [7]. Nevertheless, it is possible that, in the future, at least some of these drawbacks will be overcome by wearable implementations of different inertial technologies, currently under development, such as nano-photonic optical gyroscopes [10].

Electrogoniometers are based on strain gauges, which provide ease of use and low cost. As a drawback, the need for pacing them over joints causes possible interference with the joint’s kinematics and discomfort, due to the limited flexibility and relative size of these sensors [11,12]. Nevertheless, attempts to overcome these drawbacks are ongoing by developing new kinds of electrogoniometers made of knitted conductive yarns acting as piezo-resistive fabrics [13,14].

Electromagnetic sensors consist of wearable receiving coils that are localized relative to external emitters of magnetic fields. Whilst their accuracy is potentially high, their workspace is limited, owing to the spatial decay of the field; moreover, they are susceptible to errors, due to electromagnetic interference [15,16].

Additional wearable sensing technologies, less conventional, include piezo-resistive stretch sensors, made of conductive elastomers. They consist of planar stretchable resistors, whose resistance increases upon lengthening. The most significant advantage of this technology is represented by the ease of fabrication of large arrays of distributed, lightweight and cost-effective sensors, which can easily be integrated with garments (e.g., by screen printing) [17,18,19,20]. Nevertheless, the resistances of this type of sensor can show significant drift, due to both dimensional changes of the resistor as a result of viscoelastic creep and changes in the material’s resistivity with temperature and humidity [17,18,19,20].

This brief overview suggests that the state of the art does not offer any technology able to combine all the desirable properties of an ideal wearable sensor, i.e., high accuracy, stable response, large workspace, compact size, low weight, comfort, ease of use and low cost.

In order to explore alternative ways to monitor human kinematics with wearable systems, new investigations are being focused also on stretchable piezo-capacitive sensors (here shortly referred to as ‘capacitive’ sensors), made of dielectric elastomers. Similar to the above-mentioned stretchable piezo-resistive sensors, they too are elastomeric. However, they consist of planar deformable capacitors, which, upon stretching, can increase their electrical capacitance [21]. They are made of an elastomeric (e.g., silicone made) insulating membrane, sandwiched between two elastomeric conductive layers (e.g., made of a carbon black-silicone composite), acting as soft electrodes, so as to obtain a deformable capacitor. By using them as wearable sensors, it is possible, following a calibration, to relate variations of capacitances to changes in postures, as shown by various investigations [22,23,24,25,26,27,28,29,30,31].

Within that context, some of us proposed the use of elastomeric capacitive sensors in a wearable low-cost system aimed at detecting motions of the human trunk [32]. It consisted of modified shoulder straps carrying the sensors, in order to obtain a system easily wearable onto or under common clothes. The idea was to avoid uncomfortable, poorly practical/stable, or expensive solutions described in the literature, such as sticking (with adhesive tape) the sensors onto the skin [25,26,27,28,30], or fabricating ad-hoc garments with printed sensors [29]. Recently, we described an improved version of that system as an inclinometer, able to detect the user’s flexions, comparing its performance with those of a gyroscope and an accelerometer of a conventional IMU [33].

Here, we studied the accuracy of that system to monitor both flexions and torsions of the trunk, especially targeting the following goals:

(i) the demonstration of a strategy to calibrate the system for flexions and torsions, avoiding the limitations of typical solutions described in the literature, such as angular measurements with bulky, unpractical and/or expensive external equipment (e.g., stereophotogrammetry [22,23,24,25,26,27,28]), or pose recognitions via interpolations of data acquired from reference poses, which however do not provide accurate angular measurements [29,30,31]; to that aim, in this study, we used a wearable three-axial gyroscope;
(ii) a comparison of the sensing accuracy of a new wearable system in measuring both flexions and torsions, relative to conventional stereophotogrammetry (as the non-wearable standard technology).

## 2. Materials and Methods

### 2.1. The Wearable Sensing System

**Figure 1:**
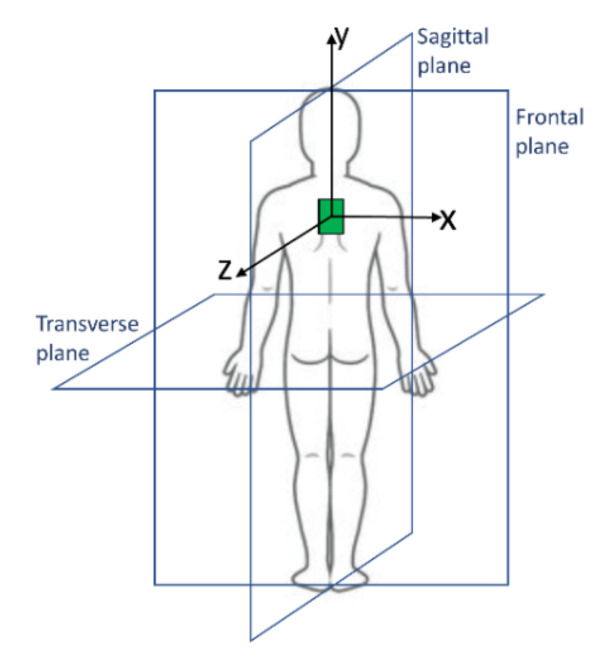
Reference planes to define the user’s motions.

**Figure 2:**
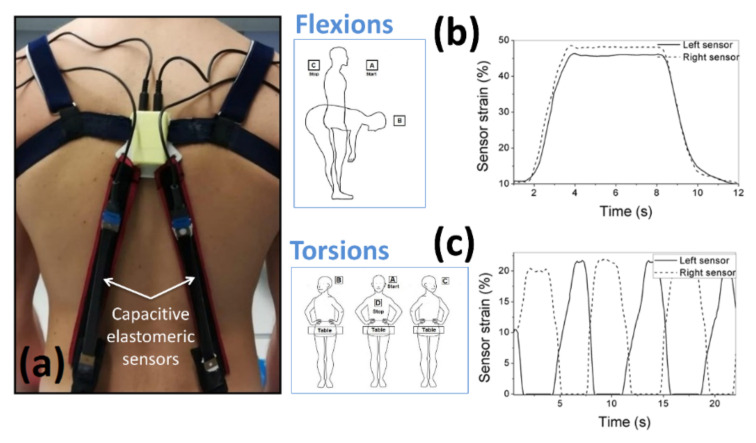
The wearable sensing system, based on a pair of capacitive elastomeric sensors (**a**), able to detect both flexions (**b**) and torsions (**c**). Flexions lengthened both the sensors, increasing their initial strain (pre-strain) to a higher value, as shown in (**b**). Torsions lengthened one of the sensors and shortened the other one, depending on the direction of motion, thereby changing their initial strain to a higher or lower value, respectively, as shown in (**c**).

The wearable system was conceived to monitor both flexions and torsions, defined as rotations of the trunk within the user’s sagittal plane and transverse plane, respectively (Figure 1). In order to detect those motions, the system consisted of modified shoulder straps, made of an elastic fabric, which supported a pair of rectangular capacitive elastomeric sensors, arranged as shown in Figure 2a.

The two sensors were identical: they were made of a silicone elastomer membrane, with carbon-based composite electrodes (Courtesy of Parker Hannifin, Mayfield Heights, OH, USA), and had at rest a length of 100 mm, a width of 15 mm and a total thickness of 1 mm (including the electrodes’ insulation coating). For each sensor, a strain along the length caused an increase in capacitance. Their sensitivity, measured as the ratio between the percentage variation of capacitance and the percentage variation of length (strain), was ~1.

One end of each sensor was connected to a plastic box, which contained wireless electronics (see the next section) and was located between the shoulder blades (Figure 2a); the other end was clamped to the user’s trousers. As a result of clamping, the sensors acquired a variable initial strain (pre-strain); for instance, in the example of Figure 2, it was 10% (as visible in Figure 2b,c, at time 0). As the pre-strain was substantially identical for the two sensors (symmetrical mounting), the two initial capacitances were substantially equal. Then, depending on the movement, the sensors could be strained in different ways, as detailed below.

Prior to starting to use the system, the user was asked to maintain an erect position for 5 s, so as to stabilize each capacitance signal. A predefined number of samples of each stabilized signal was then averaged and taken as an initial offset ($C_{offset}$), to be subtracted from the subsequent measurements. Then, the capacitive signals were processed as follows.

Any pure torsion towards one side caused the sensor on the same side to shorten (with preservation of tensioning) and the other one to lengthen. So, the strain, which was initially equal for the two sensors, was reduced for one of them and increased for the other one (Figure 2c). The same happened to their capacitances. Therefore, the absolute value and sign of the difference of capacitances were respectively indicative of the torsion’s amplitude and direction (rightwards or leftwards). Accordingly, pure torsions were easily detected by monitoring the difference between the two capacitances (after removing the initial offset), hereinafter called difference capacitance signal:

(1) $$C_{difference}\;=\;\left(\;C_{1}-C_{1,offset}\right)-\left(\;C_{2}-C_{2,offset}\right)$$

For pure flexions, the two capacitances were stretched of the same amount, such that the difference capacitance remained substantially null (*C_difference_* ≈ 0) and so it was not sensitive to the movement. However, the variation of the average signal was indicative of the flexion’s amplitude. Therefore, without changing the sensors’ layout, pure flexions were detected by monitoring the average between the two capacitances (after removing the initial offset), hereinafter called average capacitance signal:

(2) $$C_{average}=\frac{\left(\;C_{1}-C_{1,offest}\right)+\left(\;C_{2}-C_{2,offest}\right)}{2}$$

It is worth noting that, for pure torsions, the increase of one capacitance and reduction of the other one made their sum approximately null (Δ*C_average_* ≈ 0), such that their average signal was not sufficiently sensitive to the torsion.

Therefore, by continuously monitoring both the difference and the average signals, it was possible to infer whether the user was performing either a pure flexion (*C_difference_* = 0, Δ*C_average_* > 0), or a pure torsion (*C_difference_* ≠ 0, Δ*C_average_* ≈ 0), or a compound movement (*C_difference_* ≠ 0, Δ*C_average_* ≠ 0). The quantification of the movement required a calibration, which is described in the following sections.

It is important to remark that a variety of different trousers was observed to cause unpredictable shifting of the sensors’ anchoring sites, such that repeated movements of any given user could make the sensors’ stretching behavior unrepeatable. In order to minimize that problem, the volunteers involved in this study (see below) were asked to test the system in combination with the same type of elastic gym pants, adherent to the body.

### 2.2. Wireless Electronics

Continuous measurement of the two capacitances was performed with wireless electronics, consisting of a Bluno Beetle board (DFRobot, Beijing, China), equipped with a Bluetooth 4.0 module and an IMU (MPU-6050, TDK InvenSense, New York, NY, USA), which hosted a three-axial gyroscope. The gyroscope was used to both calibrate and comparatively assess the performance of the capacitive sensors (as detailed below). A 400 mAh LiPo battery ensured continuous operation for ~12 h.

The board was programmed with a custom-made algorithm for measuring the capacitance of each sensor. The algorithm continuously estimated the time constant *τ* necessary to charge the capacitor via a series resistor *R*; as a result, the capacitance *C* could be estimated as follows:

(3) C=τR

The board was also programmed to continuously extract from the gyroscope data the flexion and torsion angles, according to the following procedure. Let us consider Figure 1, where the green box schematizes the IMU (gyroscope) and *x*, *y, z* its axes. The information of interest extracted from the gyroscope consisted in the angular velocity signal within the sagittal plane, *ω_sagittal_*(*t*), i.e., relative to the axis *x*, and the angular velocity signal within the transverse plane, *ω_transverse_*(*t*), i.e., relative to the axis *y.* Those two velocities were integrated, in order to obtain the flexion and torsion angles, Φ*_flexion_* and Φ*_torsion_*, respectively:

(4) $${ф}_{flexion}=\int_{0}^{t}\omega_{sagittal}\left(t\right)dt$$

(5) $${ф}_{torsion}=\int_{0}^{t}\omega_{transverse}\left(t\right)dt$$

The capacitive and angular data were simultaneously and wirelessly transmitted to an external computer, interfaced to a Bluetooth module. In order to synchronize the signals received from the wearable system and those captured by a stereophotogrammetry system used for comparisons (as described below), a synchronization signal was generated using the computer’s sound card.

The capacitive data were used to obtain signals related to difference and average capacitances (Equations (1) and (2)), which were then translated into angular estimates, based on the calibration strategy described below.

### 2.3. Capacitive Sensors’ Calibration Using the Gyroscope

In order to relate the difference and average capacitances to the torsion and flexion angles, a calibration was necessary. However, the calibration had to be repeated each time that the system was worn, even by the same person. Indeed, while wearing the shoulder straps and securing them to the trousers, the sensors’ positions and pre-strain could vary, causing a different response to any given motion.

According to this need, we aimed to employ a calibration technique satisfying the following requirements: (i) possibility to separately achieve a calibration for flexions and a calibration for torsions; (ii) ease of implementation by the user, without assistance from others; (iii) use of compact, portable, accurate and inexpensive instrumentation; (iv) possibility to perform calibration movements of arbitrary amplitude, so as to comply with individual physical limitations.

In order to fulfill these requirements, we opted for a calibration strategy that used a wearable three-axial gyroscope integrated within a conventional IMU. The miniaturized device was easily fitted within the electronics box (Figure 2a). The idea is that this simple and cheap approach could conveniently be adopted in any capacitive sensors-based future system. It is worth noting that the idea envisages the use of the gyroscope for calibration only, excluding its use for continuous sensing over extended time; indeed, although the device remains available on board, it should not be used continuously, in order to avoid the typical errors due to drift, arising from extended integrations (as recalled in the Introduction).

The calibration process consisted of two consecutive phases, one related to pure flexions and the other one related to pure torsions, so as to obtain two independent calibrations. In particular, the user was asked to perform five cycles of pure flexions with free amplitude, followed by five cycles of pure torsions with free amplitude. Each flexion cycle consisted of flexion from the straight position, followed by an extension, reverting to the straight position. Each torsion cycle consisted of the following sequence: center–right–center–left–center. In order to minimize, during the torsions, the risk of rotations of the pelvis, as a simple strategy (targeting the highest practical viability) the user was asked to stand with the pelvis in contact with a table and then rotate the trunk about the vertical direction while keeping contact with the table (Figure 2c).

During the movements, the measurements from the capacitive sensors and gyroscope were simultaneously transmitted to the external computer, where they were processed in order to obtain the calibration. To that aim, instantaneous angular values detected with the gyroscope were plotted as a function of the corresponding instantaneous values of either the average capacitance or the difference capacitance; this allowed for achieving a calibration related to either pure flexions or pure torsions, respectively. The data, which covered the whole range of angles spanned during the five cycles of calibration movements, were linearly fitted, obtaining two calibration lines: one for pure flexions and another one for pure torsions.

### 2.4. Assessment of the Capacitive Sensors’ Accuracy

**Figure 3:**
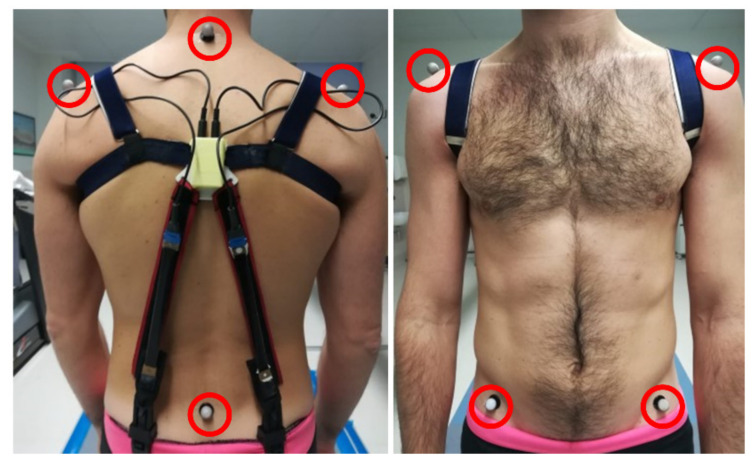
Optical markers used to enable optical tracking of the trunk’s motions via stereophotogrammetry.

The sensing accuracy of the calibrated capacitive sensors was assessed with tests on five volunteers (three females and two males, aged between 25 and 40 years), as follows. The angular signals measured by the capacitive sensors were compared with angular measurements simultaneously taken by a conventional stereophotogrammetry system (Smart DX, BTS Bioengineering Srl, Milan, Italy). To that aim, six optical markers were positioned on conventional anatomic landmarks, as shown in Figure 3, so as to enable their optical tracking by the stereophotogrammetry cameras.

**Figure 4:**
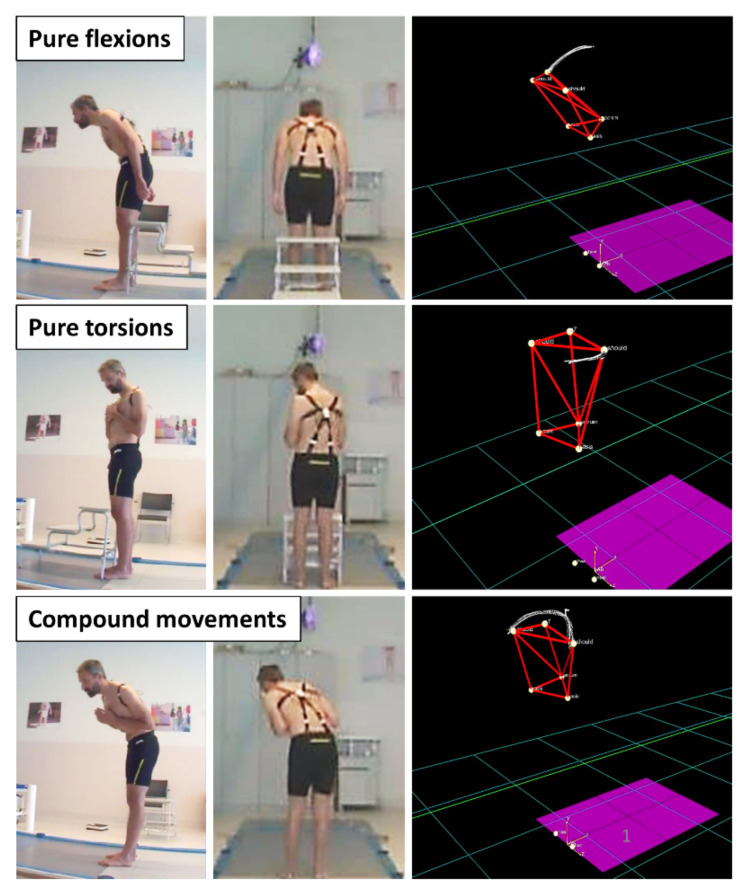
Photos and stereophotogrammetry tracking of examples of pure flexions, pure torsions and compound movements performed by a volunteer, in order to test the capacitive sensors’ accuracy relative to stereophotogrammetry.

The volunteers were asked to perform both pure flexions and pure torsions, so as to straightforwardly assess how the two movement-specific calibrations affected the sensors’ accuracy. Moreover, the volunteers were also requested to perform compound movements, consisting of uncontrolled combinations of flexions and torsions, as presented in Figure 4. This was aimed at investigating whether the two calibrations for pure movements could also be used to detect flexion and torsion components of compound movements.

For each type of motion, the accuracy relative to stereophotogrammetry was evaluated by calculating the root mean square error (*RMSE*) between the calibrated sensors’ signal *S_sens_* and the stereophotogrammetry signal *S_stereo_*:

(6) $$RMSE=\;\sqrt{\frac{\sum_{i}^{n}{\left(S_{sens,i}\;-\;S_{stereo,i}\right)}^{2}}{n}}$$

where *S_sens,i_* and *S_tereo,i_* are the *i*-th samples of the signals and *n* is the total number of samples. For each type of pure movement, the RMSE was calculated using only one type of capacitive signal (either the difference or the average one) and only one type of calibration line (either the one for pure torsions or the one for pure flexions). For compound movements, two RMSE values were calculated: one for the flexion component (using the average capacitive signal and the calibration line for pure flexions) and another one for the torsion component (using the difference capacitive signal and the calibration line for pure torsions).

## 3. Results and Discussion

### 3.1. Capacitive Sensors’ Calibration

**Figure 5:**
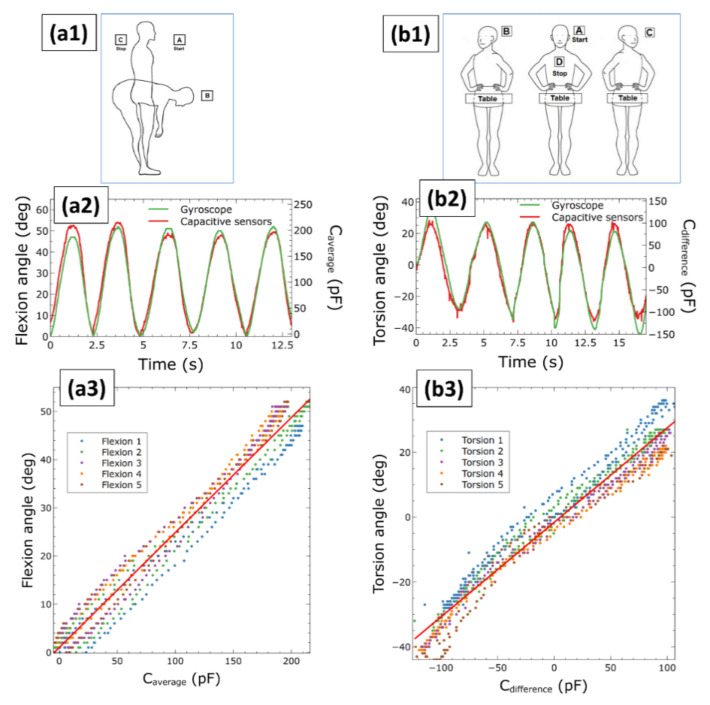
Calibration of the sensors for pure flexions (**left** panels) and pure torsions (**right** panels): calibration movements (**a1**,**b1**), examples of raw signal couples detected by the capacitive sensors and gyroscope (**a2**, **b2**) and fitting of data related to five cycles of calibration movements (indicated as flexion 1–5 and torsion 1–5) in order to obtain two calibration lines (**a3**,**b3**).

Results of the calibration are presented in Figure 5. It shows, for pure flexions and pure torsions, examples of calibration signals simultaneously obtained from the capacitive sensors (average or difference capacitance raw signal) and the gyroscope (angular signal) during five cycles of motion. The figure also shows the calibration lines obtained by fitting data collected by sampling the calibration signals, spanning five cycles.

The two independent calibrations presented in Figure 5 were used to estimate angular movements from capacitive measurements. The achieved accuracy is reported in the next section.

### 3.2. Capacitive Sensors’ Accuracy

**Figure 6:**
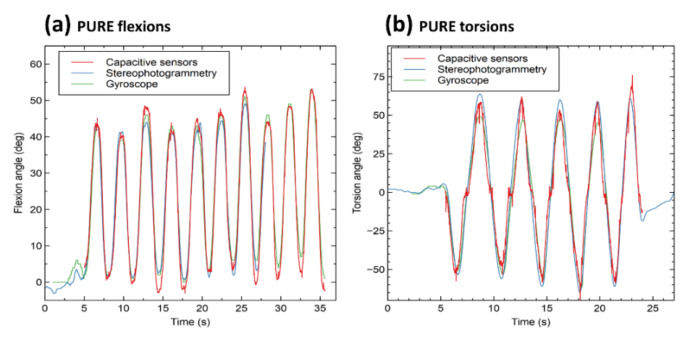
Examples of pure flexion signals (**a**) and pure torsion signals (**b**) simultaneously detected by the capacitive sensors, the stereophotogrammetry and the gyroscope.

Figure 6 presents an example of measurements. It compares signals detected by the calibrated capacitive sensors with the corresponding recordings simultaneously taken by the stereophotogrammetry, for pure flexions (Figure 6a) and pure torsions (Figure 6b). Moreover, the two signals are co-plotted with the one obtained from the gyroscope, as a further comparison.

**Figure 7:**
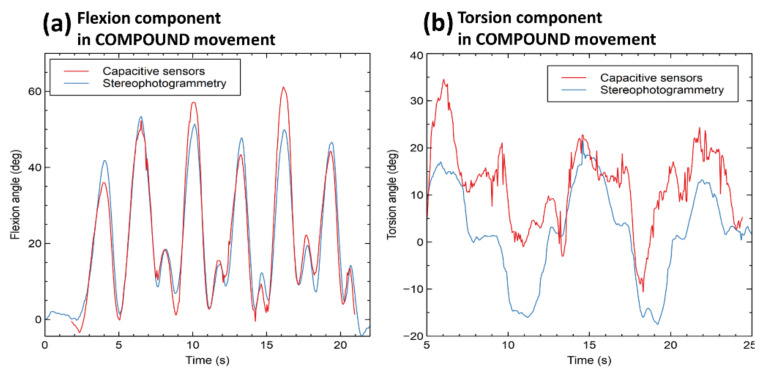
Examples of flexion component signals (**a**) and torsion component signals (**b**) simultaneously detected by the capacitive sensors and the stereophotogrammetry.

Whilst the agreement was good for pure flexions and pure torsions, it was worse for the flexion and torsion components of compound movements, as shown by the examples in Figure 7. Especially, the estimate of the torsion component was found to be less accurate (Figure 7b).

**Figure 8:**
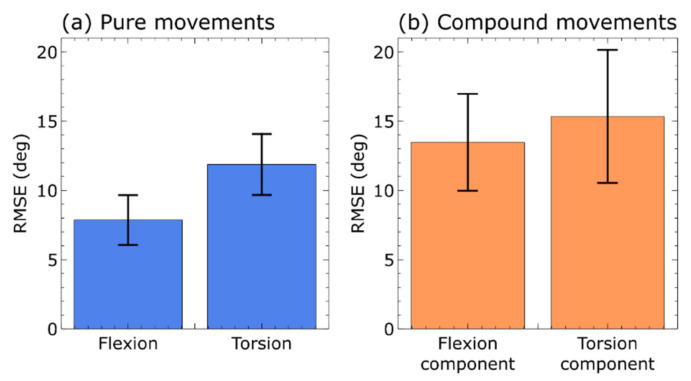
Capacitive sensors’ accuracy, as quantified by the root mean square error between the sensors’ signal and the stereophotogrammetry signal: pure movements (**a**) and compound movements (**b**). The error bars represent the standard deviation among five volunteers.

The different accuracy of the capacitive sensors for pure or compound movements is quantified in Figure 8. It presents the root mean square error relative to conventional stereophotogrammetry, calculated for pure flexions and pure torsions (Figure 8a), as well as for the flexion and torsion components of compound movements (Figure 8b). Pure flexions and pure torsions could be detected with a good agreement (average RMSE ~8° and ~12°, respectively), whilst the sensing accuracy for the flexion and torsion components in compound movements was lower (average RMSE ~13° and ~15°, respectively).

Both for pure and compound movements, the error related to flexions (or flexion components) was lower than that related to torsions (or torsion components). So, in other words, flexions could always be detected with a higher accuracy relative to torsions.

Pure movements led to lower errors as compared to compound movements. Therefore, in other words, pure flexions and torsions could always be sensed with a higher accuracy relative to the flexion and torsion components of compound movements.

The higher errors achieved with compound movements could mainly be ascribed to the following issues: (i) While performing generic motions (other than pure flexions or pure torsions), any given final posture can be reached in different ways. So, from a geometrical standpoint, any given variation of posture does not correspond to a single pair of flexion and torsion components. However, during stereophotogrammetry-based motion tracking, only a single pair of components is extracted: it corresponds to the convention used to define the Euler angles (choice of the axes about which the rotations are made and their sequence). Even for the capacitive sensors, only a single pair of components were extracted; however, they consisted of a different couple, as they were obtained from the calibration for pure flexions and pure torsions. Therefore, the compared data related to compound movements are affected by a systematic difference between stereophotogrammetry and capacitive sensors. (ii) The second source of error was occasional, rather than systematic and consisted of unintentional rotations of the pelvis, as the latter was not immobilized (Figure 4). Whilst those rotations were properly captured by the stereophotogrammetry via the lower markers (Figure 3), they were not taken into account by processing the capacitive signals, as they had been calibrated with two independent pure movements. (iii) Another possible source of occasional error was represented by the fact that, during a compound movement, the capacitive sensors could be stretched in different ways, such that one or both of them could occasionally and partially adhere to the user’s trunk. The occurring friction with the body could temporarily alter the stretching behavior and therefore also the related variation of capacitance.

## 4. Conclusions and Future Developments

This work presented an easy-to-use and cost-effective wearable system for continuous monitoring of flexions and torsions of the human trunk, either as pure or as compound movements. It used elastomeric capacitive stretch sensors arranged on shoulder straps. A simple and fast calibration strategy was implemented, using an onboard tri-axial gyroscope. The sensing accuracy relative to stereophotogrammetry corresponded in general to an average RMSE not higher than 15°.

While this system showed the benefits of using capacitive elastomeric sensors to monitor two degrees of freedom (flexion and torsion), greater advantages are expected to arise from more extensive use of such wearable sensors, especially in terms of large arrays. Indeed, this type of sensor is expected to show its highest potential for monitoring a large number of degrees of freedom, via dense arrays of small sensors able to implement sensing distributed on the body. This is motivated by the higher simplicity and lower cost than they would offer, as an alternative to the use, for instance, of a large number of IMUs. With respect to them, an array of capacitive sensors not only would avoid the typical problems recalled in the Introduction, but also could be distributed on the body more easily and more conformably, e.g., by printing many thin, lightweight and stretchable sensors on garments. This strategy has already been demonstrated in some cases, such as the prototype glove described by Glauser et al. [29] and the commercial gloves produced by StretchSense [34]. Such examples have strong roots in previously described distributed large arrays of wearable elastomeric sensors, based on the piezo-resistive effect [17,18,19,20].

Nevertheless, the development of large arrays of distributed stretchable capacitive sensors would raise challenges to achieve adequate calibrations of the system. Indeed, as the number of degrees of freedom increases, the complexity of calibrating each of them with an easy-to-use, miniaturized and low-cost technology (as targeted in this work) would rapidly scale up, both from a technological and a methodological point of view. Therefore, for such systems, the goal considered in this work of monitoring a certain number of degrees of freedom should give room to a different way of conceiving the role of a wearable system for distributed sensing: instead of measuring angles, the system should recognize poses (postures or gestures), without knowledge of the concerned body segments’ angles. The poses would correspond to different states of body portions, such as a set of postures of the trunk or a set of gestures of the hand and they could be classified according to different aims, such as differentiating healthy and risky postures or interpreting a gesture language. This approach would take advantage of the use of machine learning algorithms, in order to train, for instance, a neural network. This has been demonstrated in the past for large distributed arrays of piezo-resistive elastomeric sensors [17,18,19,20] and ongoing work is confirming equal benefits for arrays of piezo-capacitive elastomeric sensors [29,34].

## Data Availability

The data presented in this study are available on request from the corresponding author. The data are not publicly available due to restrictions on privacy.
